# P-2095. Analyzing the Impact of an Intensive Care Coordination Program Based on Acuity Scores in People with HIV

**DOI:** 10.1093/ofid/ofaf695.2259

**Published:** 2026-01-11

**Authors:** Smitha Gudipati, Johnny Zakhour, Victoria Warzocha, Nicholas F Yared, Brianna Hohmann, Shannon Payne, Jamie G Joseph, Indira Brar

**Affiliations:** Henry Ford Health System, Detroit, MI; Henry Ford Health, Detroit, Michigan; Henry Ford Health, Detroit, Michigan; Henry Ford Health System, Detroit, MI; Henry Ford Health, Detroit, Michigan; Henry Ford Health, Detroit, Michigan; Henry Ford Health/MSU, Royal Oak, Michigan; Henry Ford Hospital, Detroit, Michigan

## Abstract

**Background:**

Social Determinants of Health (SDOH) impact virologic suppression in people with HIV (PWH). Care Coordination Program (CCP) is an intensive comprehensive program that addresses SDOH to improve care in PWH with a history of suboptimal outcomes. Success of CCP at Henry Ford Health (HFH) was assessed based on PWH’s acuity scores, which are used to identify PWH who are at higher risk for negative health outcomes related to SDOH. The difference in acuity scores between those who had a positive outcome (graduated) after being enrolled in CCP versus a negative outcome (discharged) was assessed in this study.

**Figure:**
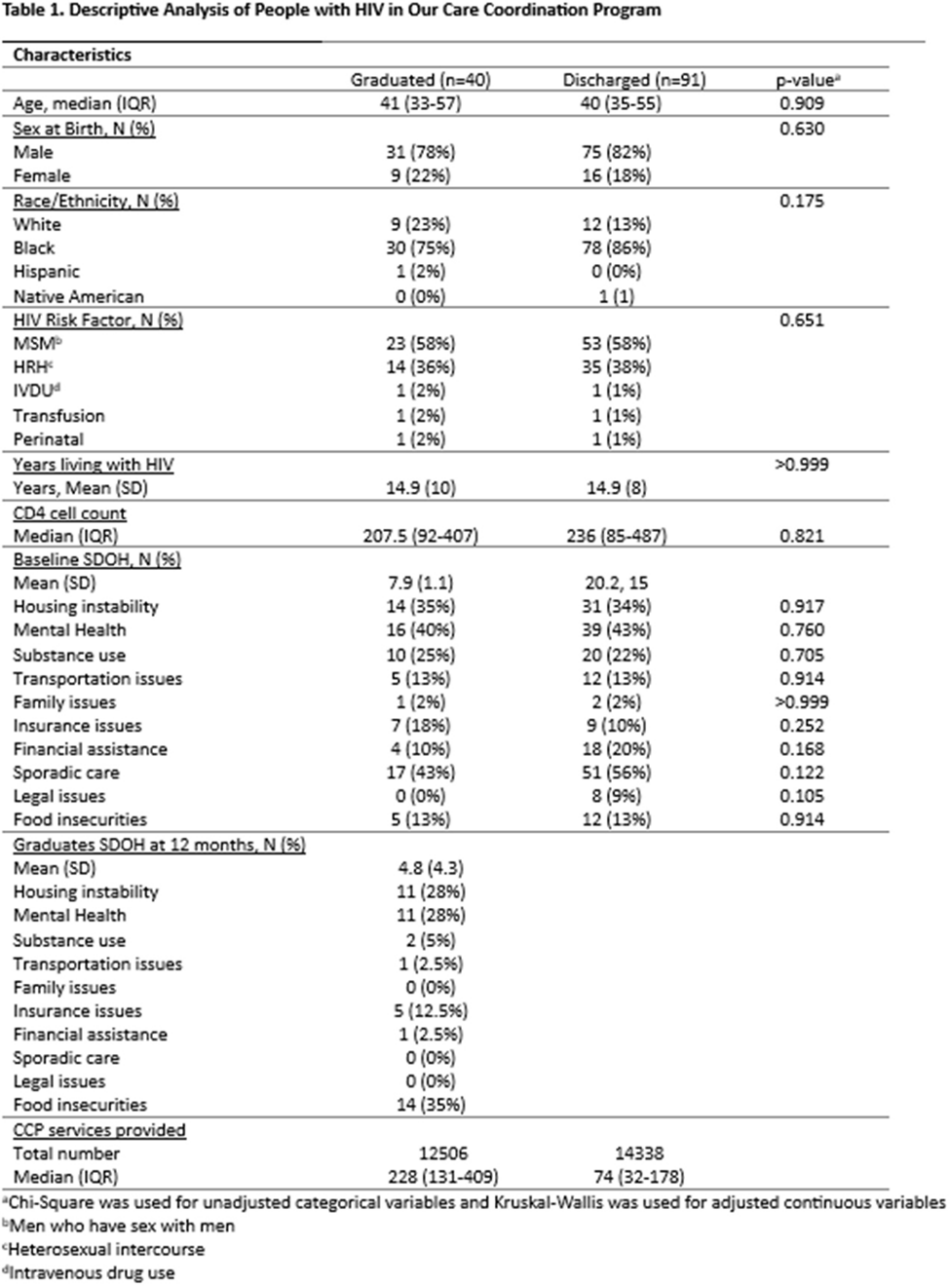

**Figure:**
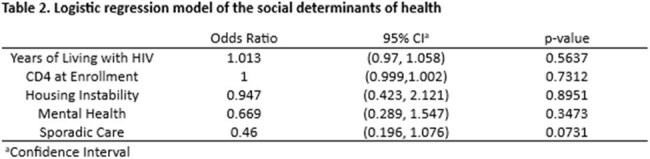

**Methods:**

A retrospective study was conducted on PWH in CCP at HFH from 2019 to 2024. PWH were categorized into those that graduated or discharged from CCP after one year of services. An acuity score, which determined severity of SDOH barriers that impact patient care, was assigned to each patient, based on Michigan Department of Health and Human Service’s Ryan White Acuity Scale. PWH in CCP were supported to overcome SDOH needs for 1 year. Graduation was defined as achieving HIV-1 viral load (VL) < 200 copies/mL at 1 year. If viral suppression was not achieved at 1 year or PWH did not engage in the program, they were discharged.

**Results:**

A total of 131 PWH were enrolled in CCP; 41 graduated, 91 were discharged. Median age was 40 (IQR:34-55); 82.4% were Black; 80.9% were male, and median VL and CD4 cell count were 83,920 copies/ml (IQR:265-77069) and 231 cells/mm^3^ (IQR:81-483). Median acuity score at enrollment in CCP was 45 (IQR:34.5-54.5) and 37.5 (IQR: 33.5-47) (SD=9) in the graduating and discharge groups respectively. After 1 year of CCP, median acuity scores were 27 (IQR:22-33) in the graduating group and 37 (IQR: 29-43) in the discharged group (Table 1). On average, we observed a higher rate of sporadic care in the discharged group (OR 0.46; 95% CI (0.196, 1.076); p=0.0731)) (Table 2).

**Conclusion:**

PWH who had a reduction in acuity scores as a result of CCP had a 45% success rate in achieving viral suppression. Among those discharged, there was no significant change in their acuity scores, and these PWH are more likely to have negative health outcomes due to viremia. By identifying SDOH issues in this group of PWH, healthcare providers can implement targeted interventions to address these factors.

**Disclosures:**

Indira Brar, MD, Gilead: Advisor/Consultant|Gilead: Grant/Research Support|Gilead: Honoraria|ViiV Healthcare: Advisor/Consultant|ViiV Healthcare: Grant/Research Support|ViiV Healthcare: Honoraria

